# Effects of *Acremonium terricola* Culture on Lactation Performance, Immune Function, Antioxidant Capacity, and Intestinal Flora of Sows

**DOI:** 10.3390/antiox13080970

**Published:** 2024-08-09

**Authors:** Zhirong Chen, Lixia Xiao, Qian Sun, Qiangqiang Chen, Weidong Hua, Jinzhi Zhang

**Affiliations:** 1 College of Animal Science, Zhejiang University, Hangzhou 310058, China; chenzhirong169@zju.edu.cn (Z.C.); 22017065@zju.edu.cn (L.X.); 22217091@zju.edu.cn (Q.S.); 21917003@zju.edu.cn (Q.C.); 2 Institute of Animal Husbandry and Veterinary Medicine, Zhejiang Academy of Agricultural Sciences, Hangzhou 310021, China; huaweidong833@sohu.com

**Keywords:** *Acremonium terricola*, sows, lactation performance, immune, antioxidant, intestinal flora

## Abstract

This study aimed to determine the effects of different doses of *Acremonium terricola* culture (ATC) on lactation performance, immune function, antioxidant capacity, and intestinal flora of sows. Forty-five Landrace sows (3–6 parity) were randomly assigned to the following three treatments from 85 days of gestation to 21 days after farrowing: a control diet (CON, basal diet), a low-dose *Acremonium terricola* culture diet (0.2% ATC, basal diet + 0.2% ATC), and a high-dose *Acremonium terricola* culture diet (0.4% ATC, basal diet + 0.4% ATC). Compared with the CON group, the supplementation of 0.2% ATC increased the average daily milk yield of sows by 4.98%, increased milk fat, total solids, and freezing point depression on day 1 postpartum (*p* < 0.05), increased serum concentration of Triiodothyronine, Thyroxin, and Estradiol on day 21 postpartum (*p* < 0.05). Compared with the CON group, the supplementation of 0.4% ATC increased the average daily milk yield of sows by 9.38% (*p* < 0.05). Furthermore, the supplementation of 0.2% ATC increased serum concentration of IgG, IgM, and IFN-γ, CD4 on day 1 postpartum (*p* < 0.05) and increased serum concentration of immunoglobulin A ( IgA), immunoglobulin G (IgG), immunoglobulin M ( IgM), complement 3 (C3), cluster of differentiation 4 (CD4), cluster of differentiation 8 (CD8), interferon-γ (IFN-γ) on day 21 postpartum (*p* < 0.05), while the supplementation of 0.4% ATC reduced serum concentration of IL-2 on day 21 postpartum (*p* < 0.05). Moreover, the supplementation of 0.4% ATC significantly increased serum concentration of catalase (CAT) (*p* < 0.05). Additionally, the supplementation of ATC affected the relative abundance of the intestinal flora at different taxonomic levels in sows and increased the abundance of beneficial bacteria such as in the *norank_f__Eubacterium_coprostanoligenes group*, *Eubacterium_coprostanoligenes group*, and *Lachnospiraceae_XPB1014 group* of sows, while reducing the abundance of harmful bacteria such as *Phascolarctobacterium* and *Clostridium_sensu_stricto_1*. These data revealed that the supplementation of ATC during late gestation and lactation can improve lactation performance, immune function, antioxidant capacity, and the gut microbiota. Compared with supplementation of 0.4% ATC, 0.2% ATC enhances the levels of thyroid-related hormones, specific antibodies, and cytokines in serum, promotes the diversity of beneficial gut microbiota, beneficial bacteria in the intestine, reduces the population of harmful bacteria, and thereby bolsters the immunity of sows. Hence, 0.2% ATC is deemed a more optimal concentration.

## 1. Introduction

In large-scale pig production, sows and piglets are the key determining the production level and economic benefits of pig farms. The gestation and lactation periods of sows and the preweaning period of the newborn piglets are core stages of feed management in large-scale pig production [1]. During pregnancy, the physiological metabolism and immunity of sows undergo drastic changes to ensure the implantation and development of embryos and the completion of pregnancy [2]. In the middle and later stages of pregnancy, the levels of tumor necrosis factor-α (TNF-α), interleukin-6 (IL-6), and other pro-inflammatory factors in the blood of sows are significantly increased [3] and are closely related to many complications, including constipation, abortion, and intrauterine growth delay [4,5]. In addition, the expression of tight junction protein ZO-1 in the gut of sows is reduced, while LPS entering circulation through the intestinal barrier is increased, and the increased concentration of bacterial endotoxin in circulation can lead to metabolic endotoxemia, which is a potential inflammatory mediator [6]. Sows are also very susceptible to diseases during the perinatal period, such as metabolic disorders and systemic inflammation, resulting in changes in their voluntary feed intake and endocrine homeostasis, which have a negative impact on the reproductive performance of sows [7]. Postpartum dyslactation syndrome is a perinatal disease of sows, which will cause large economic losses in pig breeding and reduce litter size, parity number, and health status rate of sows [8]. At the same time, studies have also shown that the intestinal microbiota composition of the female changes during pregnancy, which may contribute to maternal metabolism and immune adaptation [9,10].

In lactation, sow milk is an important source of nutrition for piglets. For example, colostrum provides energy for body temperature regulation and the growth of newborn piglets, provides passive immunity for defense against pathogens, and stimulates the growth and development of tissues and organs, especially the gastrointestinal tract [11]. Colostrum intake is critical for piglet survival in the first few days after birth and for piglet health and growth performance before weaning [11], and it is positively correlated with piglet weight at weaning and at the beginning and end of the fattening period, and negatively correlated with piglet mortality during the nursery period [12]. Breeding for the specific physiological state of sows is a key factor in improving animal health and welfare as well as pig productivity [13]. *Cordyceps sinensis*, renowned for its extensive pharmacological effects—including anti-inflammatory, antioxidant, anti-tumor, and immune-enhancing properties—is widely used in China as a nourishing food and supplement with broad-spectrum medicinal value [14,15]. Its bioactive components, such as cordycepin and polysaccharides, are primarily responsible for these effects [16]. However, due to its scarcity and high cost, *Cordyceps sinensis* is typically reserved for human consumption and is not used as an additive in feed. *Acremonium terricola* is a parasite isolated from *Cordyceps gunnii*; artificial solid fermentation is then used to obtain *Acremonium terricola* culture (ATC). ATC is a new feed additive with bioactive ingredients that are the same as those of *C. gunnii*, such as *Cordyceps* polysaccharides, cordycepin, cordycepic acid, ergosterol, and amino acids [17]. *Cordyceps* polysaccharide derived from ATC in the diet is a water-soluble polysaccharide polymer, and some studies have reported that *Cordyceps* polysaccharide plays a crucial role in manipulating the gastrointestinal microbiota by providing nutrients for beneficial microorganisms [18,19]. As previously reported, ATC has a wide range of biological functions, such as antioxidant action, immune regulation, and anti-inflammatory activity. In addition, ATC also has the effect of improving milk performance of cows [20]. Therefore, the main objective of our study was to investigate the effects of ATC supplementation on the lactation performance, immunity, antioxidant competence, and intestinal health of sows.

## 2. Materials and Methods

### 2.1. Ethics Statement, Experiment Design, and Management

Zhejiang University’s Animal Protection Institution and Use Committee (Zhejiang, Hangzhou, China) granted its approval to carry out experiments involving animals. The ethics committee number of this research institution is ZJU20170466.

Forty-five pregnant Landrace sows with similar parity, weight, and farrowing date were randomly divided into 3 groups, the control group (CON), 0.2% *Acremonium terricola* culture group (0.2% ATC), 0.4% *Acremonium terricola* culture group (0.4% ATC), with 15 sows in each group. The experiment period began from 85 days of gestation to 21 days postpartum. The sows were fed twice a day at 08:00 and 16:00 during the entire experimental phase. They were feed-restricted for 3 days before delivery and began to freely feed and drink postpartum. Within 24 h postpartum, the litter size was standardized to approximately 11 piglets by cross-fostering in all groups. During the trial period, all test sows and piglets were managed according to the management protocol and immunization program of institute of Animal Husbandry and Veterinary Medicine, Zhejiang Academy of Agricultural Sciences. The diet components and nutrient composition of sows during the whole trial period are shown in Table 1.

### 2.2. Sample Collection

The daily feed intake of the sows was recorded postpartum. According to the fact that the milk yield of sows was 4 times the litter gain of piglets, the piglets were weighed, and the litter weight was recorded every 7 d (1–7 d, 8–14 d and 15–21 d) after the sows gave birth, and the average daily milk yield of sows was recorded every 7 d (1–7 d, 8–14 d and 15–21 d). On 1 d and 21 d postpartum, 6 sows were randomly selected from each group, and the milk samples were collected by ear vein injection of oxytocin and stored in a refrigerator at −20 °C. Blood samples were collected through the anterior vena cava. The serum was separated after centrifugation at 3000 r/min at 4 °C for 10 min and stored in a −80 °C refrigerator for testing. Fresh fecal samples of sows were collected by rectal touch method and stored in liquid nitrogen at −80 °C.

### 2.3. Milk Composition

Milk samples were thawed at 4 °C and mixed thoroughly before analysis, and milk composition was determined using the MilkoScon FT 120 (type 71200) (FOSS, Hillerød, Denmark) machine. Milk composition including fat, protein, lactose, total solids, solid nonfat, density, acidity, freezing point depression, free fatty acid, and citric acid concentrations were determined.

### 2.4. Serum Parameters

Serum samples were thawed at 4 °C and mixed thoroughly before analysis. The serum immune parameters including prolactin (PRL), growth hormone (GH), insulin growth factor 1 (IGF-1), insulin (INS), triiodothyronine (T3), thyroxine (T4), estradiol (E2); total protein (TP), albumin (ALB), glucose (GLU), cholesterol (TCH), urea nitrogen (BUN), creatinine (CRE), lactate dehydrogenase (LDH), alkaline phosphatase (AKP), glutamic oxalic aminotransferase (GOT), glutamic pyruvic aminotransferase (GPT); immunoglobulin A(IgA), immunoglobulin G (IgG), immunoglobulin M(IgM), interleukin-2 (IL-2), interleukin-4(IL-4), complement 3 (C3), complement 4 (C4), tumor necrosis factor-α (TNF-α), cluster of differentiation 4 (CD4), cluster of differentiation 8 (CD8), interferon-γ (IFN-γ). Serum antioxidant parameters including superoxide dismutase (SOD), malondialdehyde (MDA), glutathione peroxidase (GSH-Px), total antioxidant capacity (T-AOC), and catalase (CAT) were measured by the commercial kits (Nanjing Jiancheng Bioengineering Institute, Nanjing, China) according to the instructions.

### 2.5. DNA Extraction, PCR Amplification and Sequencing

Total genomic DNA was extracted from the sow fecal samples using the E.Z.N.A.^®^ Soil DNA Kit (Omega Bio-tek, Norcross, GA, USA) and the DNA integrity was tested by 1% agarose gel electrophoresis, after which the upstream primer 338F—ACTCCTACGGGAGGCAGCAG—and the downstream primer 806R—GGACTACHVGGGTWTCTAAT—we used to amplify the V3–V4 hypervariable region of the bacterial 16S rRNA gene. The PCR amplification process of the 16S rRNA gene was as follows: initial denaturation at 95 °C for 3 min, denaturation cycle at 95 °C for 30 s, annealing at 55 °C for 30 s, extension at 72 °C for 45 s, and extension at 72 °C for 10 min. PCR products were detected using 2% agarose gel electrophoresis and purified using the AxyPrep DNA Gel Recovery Kit (Axygen, Union City, CA, USA) according to the manufacturer’s instructions. PCR products were quantified with Quantus™ Fluorometer (Promega, Madison, WI, USA) and sequenced per sample, then mixed in proportion. After quantification, the PCR products were sequenced on the Illumina MiSeq PE300 (Illumina, San Diego, CA, USA) platform.

### 2.6. Statistical Analysis

The collected data were analyzed by one-way ANOVA analysis using SPSS software 27.0 and multiple comparative analysis using Duncan’s test. The Kruskal–Wallis test was used to analyze the differences in intestinal flora at the phylum and genus levels before being corrected by Welch’s (uncorrected) method. Additionally, the relationships between the gut microbiota and serum immune parameters were explored by Spearman’s correlation analysis. Clustering correlation heatmap and network with signs were performed using the OmicStudio tools at https://www.omicstudio.cn (accessed on 20 January 2024). A significant difference was declared at *p* < 0.05.

## 3. Results

### 3.1. Daily Feed Intake of Lactation Sows

The effects of ATC on daily feed intake of the lactation sows are presented in Table 2. The 0.2% ATC group increased daily feed intake of the lactation sows at day 8–14 postpartum (*p* < 0.05), neither 0.2% ATC group nor 0.4% ATC group had no significant effects on the average daily feed intake of sows at day 1–21 postpartum.

### 3.2. Average Daily Milk Yield of Sows

Effects of ATC on average daily milk yield of sows are presented in Table 3. The 0.2% ATC group and 0.4% ATC group increased the average daily milk yield at day 1–21 postpartum by 4.98% and 9.38%, respectively, but the difference was not significant.

### 3.3. Milk Composition

The effects of ATC on milk composition of sows are presented in Table 4. No significant differences were observed in milk composition of the sows on day 1 postpartum. Compared with CON, the 0.2% ATC group significant increases in milk fat, total solids, and freezing point depression on day 21 postpartum (*p* < 0.05). The 0.4% ATC group had no significant difference in the milk composition of sows on day 21 postpartum.

### 3.4. Serum Hormone Parameters

**Figure 1:**
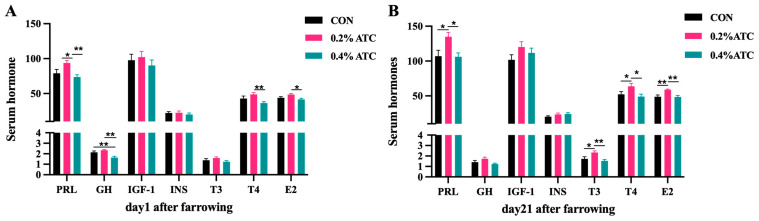
Effects of ATC on serum hormone indexes of sows on day 1 (**A**) and day 21 (**B**) postpartum. Note: The unit of serum indexes of sows as fallowing: PRL, GH, IGF-1, T3, T4: ng/mL, INS: mIU/L, E2: ng/L. The “*” above the bar graph of serum indexes indicates significant differences (*p* < 0.05) and “**” indicates highly significant differences (*p* < 0.01).

The effects of ATC on serum hormone parameters of sows are presented in Figure 1A,B. On day 1 postpartum, the 0.2% ATC group had increased serum content of PRL (*p* < 0.05), and the 0.4% ATC group decreased serum content of GH (*p* < 0.05). Compared with the 0.4% ATC group, the 0.2% ATC group significantly increased the content of T4 (*p* < 0.01) and E2 (*p* < 0.05). On day 21 postpartum, a significant increase in serum content of PRL, T3, T4, and E2 were observed in the sows fed 0.2% ATC when compared with those fed CON (*p* < 0.05). Compared to the 0.4% ATC group, the 0.2% ATC group had significantly increased content of PRL, T4 (*p* < 0.05), T3 and E2 (*p* < 0.01).

### 3.5. Serum Biochemical Parameters

**Figure 2:**
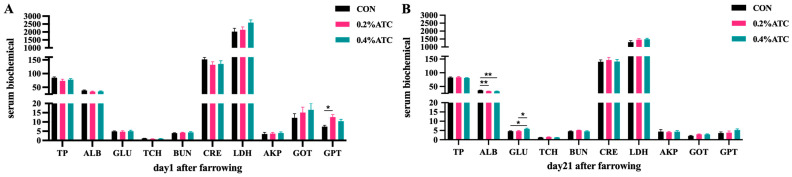
Effects of ATC on serum biochemical indexes of sows on day 1 (**A**) and day 21 (**B**) postpartum. Note: The unit of serum indexes of sows as fallowing: TP, ALB: g/L, GLU, TCH, BUN: mmol/L, CRE: μmol/L, LDH, GOT, GPT: U/L. The “*” above the bar graph of serum indexes indicates significant differences (*p* < 0.05) and “**” indicates highly significant differences (*p* < 0.01).

Effects of ATC on serum biochemical parameters of sows are presented in Figure 2A,B. On day 1 postpartum, the 0.2% ATC group had increased serum content of GPT (*p* < 0.05). On day 21 postpartum, the 0.2% group and 0.4% ATC group had highly significant decreased serum content of ALB (*p* < 0.01). Compared to the CON group, the 0.2% ATC group and 0.4% ATC group had increased serum content of GLU (*p* < 0.05).

### 3.6. Serum Immune Parameters

**Figure 3:**
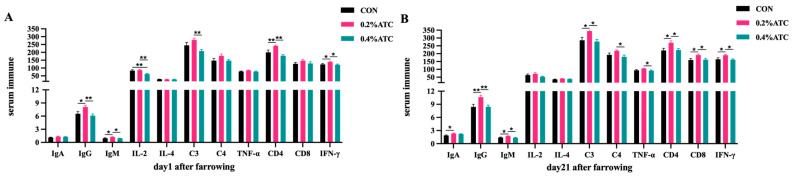
Effects of ATC on serum immune indexes of sows on day 1 (**A**) and day 21 (**B**) postpartum. Note: The unit of serum indexes of sows as following: AKP: King’s Unit/0.1 L; IgA, IgG, IgM, C3, C4: μg/mL, IL-2, IL-4, TNF-α, IFN-γ: pg/mL, CD4, CD8: IU/mL. The “*” above the bar graph of serum indexes indicates significant differences (*p* < 0.05) and “**” indicates highly significant differences (*p* < 0.01).

Effects of ATC on serum immune parameters of sows are presented in Figure 3A,B. On day 1 postpartum, compared to CON, the 0.2% ATC group had increased serum content of IgG, IgM, IFN-γ (*p* < 0.05) and extremely significant increased contents of IL-2, CD4 (*p* < 0.01). Compared to the 0.4% ATC group, the 0.2% ATC group had increased serum content of IgM and IFN-γ (*p* < 0.05) as well as extremely significant increased content of IgG, IL-2, C3, CD4 (*p* < 0.01). Compared to CON, the 0.4% ATC group had extremely significant increased serum content of IL-2 (*p* < 0.01). On day 21 postpartum, compared with CON, the 0.2% ATC group had increased serum content of IgA, IgM, C3, CD4, CD8, IFN-γ (*p* < 0.05) and extremely significant increased content of IgG (*p* < 0.01). Compare with the 0.4% ATC group, the 0.2% ATC group had increased serum content of IgM, C3, C4, TNF-α, CD4, CD8, IFN-γ, and extremely significant increased content of IgG (*p* < 0.01).

### 3.7. Serum Antioxidant Parameter

**Figure 4:**
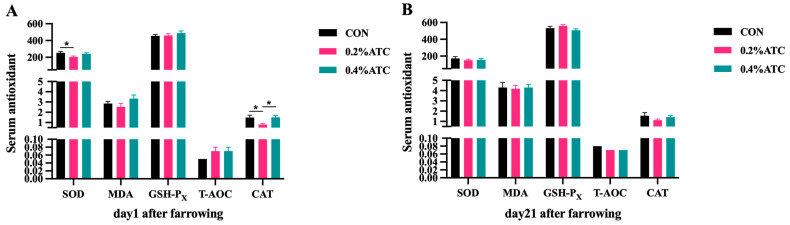
Effects of ATC on serum antioxidant indexes of sows on day 1 (**A**) and day 21 (**B**) postpartum. Note: The unit of serum indexes of sows as fallowing: SOD, GSH-PX, CAT unit: U/mL; MDA unit: nmol/mL; T-AOC unit: mmol/L. The “*” above the bar graph of serum indexes indicates significant differences (*p* < 0.05).

Effects of ATC on serum antioxidant parameters of sows are presented in Figure 4A,B. On day 1 postpartum, the 0.4% ATC group significantly increased the serum content of CAT compared to the 0.2% ATC group (*p* < 0.05), but compared with CON, the serum content of SOD decreased in the supplementation of 0.2% ATC (*p* < 0.05). On day 21 postpartum, there were no significant differences in the antioxidant indexes in the serum of sows between the different treatment group (*p* > 0.05).

### 3.8. Fecal Microbiota

**Figure 5:**
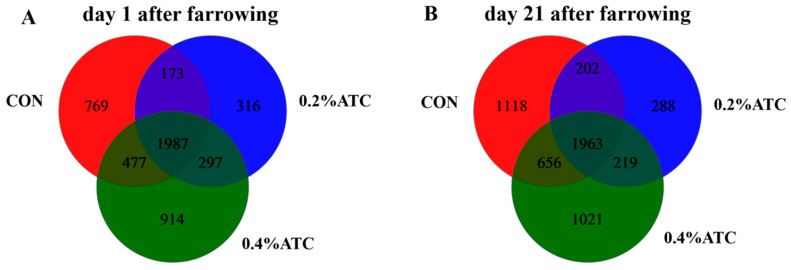
A Venn diagram of common and unique OTUs in the gut microbiota of sows. (**A**) day 1 postpartum. (**B**) day 21 postpartum.

In order to explore the effect of ATC on the intestinal microflora of sows, 16S rRNA sequencing was performed on 36 female pig fecal samples, and 6648,120 sequences were generated. Based on 97% sequence similarity, a total of 4933 OTUs were identified on day 1 postpartum, as shown in Figure 5A. Among these, 1987 OTUs were shared across the three groups, constituting the core OTUs and representing 40.28% of the total. The unique OTUs were distributed as follows: 769 in the CON group, 316 in the 0.2% ATC group, and 914 in the 0.4% ATC group. On day 21 postpartum, as depicted in Figure 5B, a total of 5467 OTUs were identified. Of these, 1963 were core OTUs shared among the three groups, accounting for 35.91% of the total. The unique OTUs were 1118 in the CON group, 288 in the 0.2% ATC group, and 1021 in the 0.4% ATC group.

**Figure 6:**
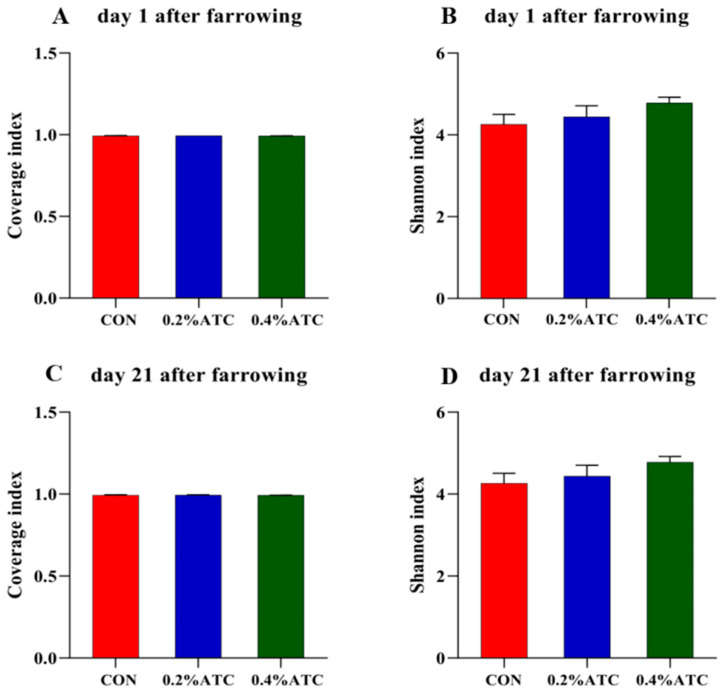
Microbiota alpha diversity comparison of sows. Alpha diversity of fecal microbial community determined by Coverage index of sows. (**A**) on day 1 postpartum. (**C**) on day 21 postpartum. Alpha diversity of fecal microbial community is determined by Shannon index of sows. (**B**) on day 1 postpartum. (**D**) on day 21 postpartum.

As shown in Figure 6A,C, the coverage value of the intestinal flora of the sows on day 1 and day 21 postpartum was above 0.99, indicating high coverage of sequencing results, which could better reflect the distribution of the intestinal flora of the sows. As shown in Figure 6B,D, there was no significant difference in the Shannon index among the different treatment groups in the alpha-diversity index of the intestinal flora of the sows on day 1 and day 21 postpartum.

**Figure 7:**
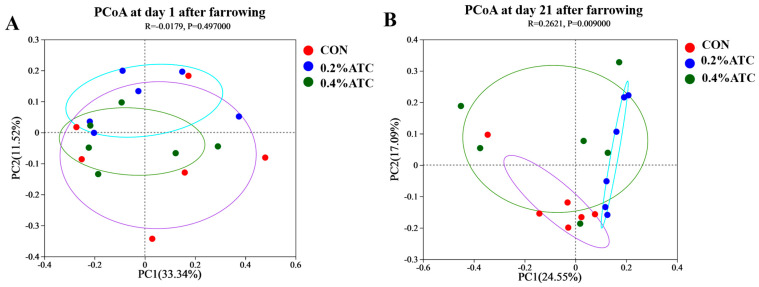
Microbiota beta diversity comparison of sows. (**A**) day 1 postpartum. (**B**) day 21 postpartum.

As shown in Figure 7A,B, principal coordinate analysis (PCoA) based on Bray–Curtis dissimilarity revealed that there was a clear separation of the microbial community among the CON and 0.2% ATC group on day 21 postpartum, indicating a shift in gut microbial communities (*p* < 0.05).

**Figure 8:**
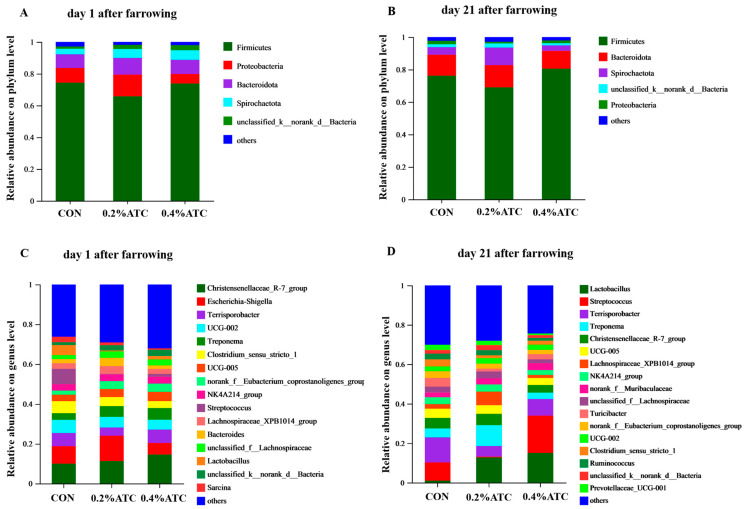
Taxonomic distribution of bacterial phyla and genera of gut microbiota of sows. Taxonomic distribution of bacterial phyla of gut microbiota of sows. (**A**) on day 1 postpartum. (**B**) on day 21 postpartum. Taxonomic distribution of bacterial genera of gut microbiota of sows. (**C**) on day 1 postpartum. (**D**) on day 21 postpartum.

Subsequently, the effects of ATC on gut microbial composition in sows were investigated. As shown in Figure 8A,B, at the phylum level, the dominant phyla were *Firmicutes* and *Proteobacteria* on day 1 postpartum, accounting for 80.92%, and the dominant phyla were Firmicutes and Bacteroidota on day 21 postpartum, accounting for 87.75%. As shown in Figure 8C,D, On day 1 postpartum, the top five genera were *Christensenellaceae_R-7 group*, *Escherichia-Shigella*, *Terrisporobacter*, *UCG-002* and *Treponema.* On day 21 postpartum, the top five genera were *Lactobacillus*, *Streptococcus*, *Terrisporobacter*, *Treponema*, *Christensenellaceae_R-7 group*.

**Figure 9:**
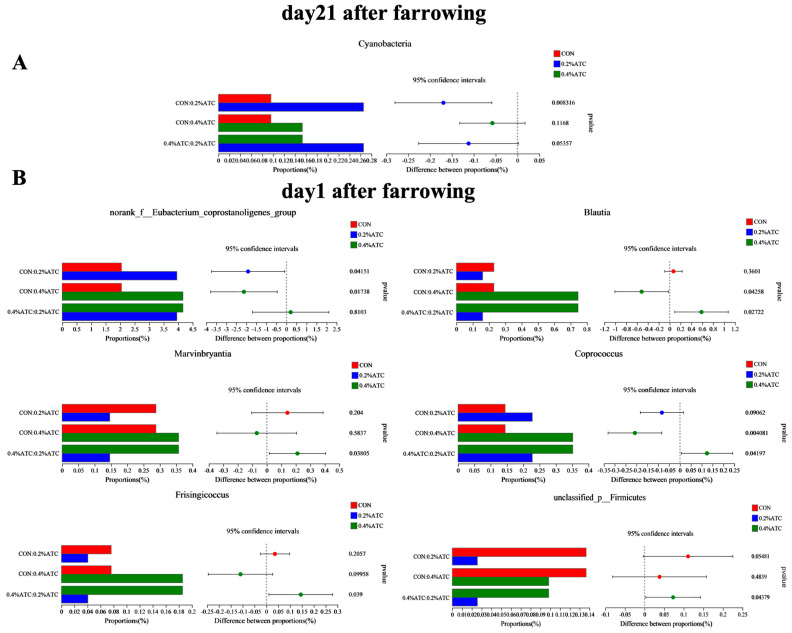
Changes in bacterial phyla and genera of gut microbiota of sows. (**A**) Changes in bacterial phyla of gut microbiota of sows on day 21 postpartum. (**B**) Changes in bacterial genera of gut microbiota of sows on day 1 postpartum.

**Figure 10:**
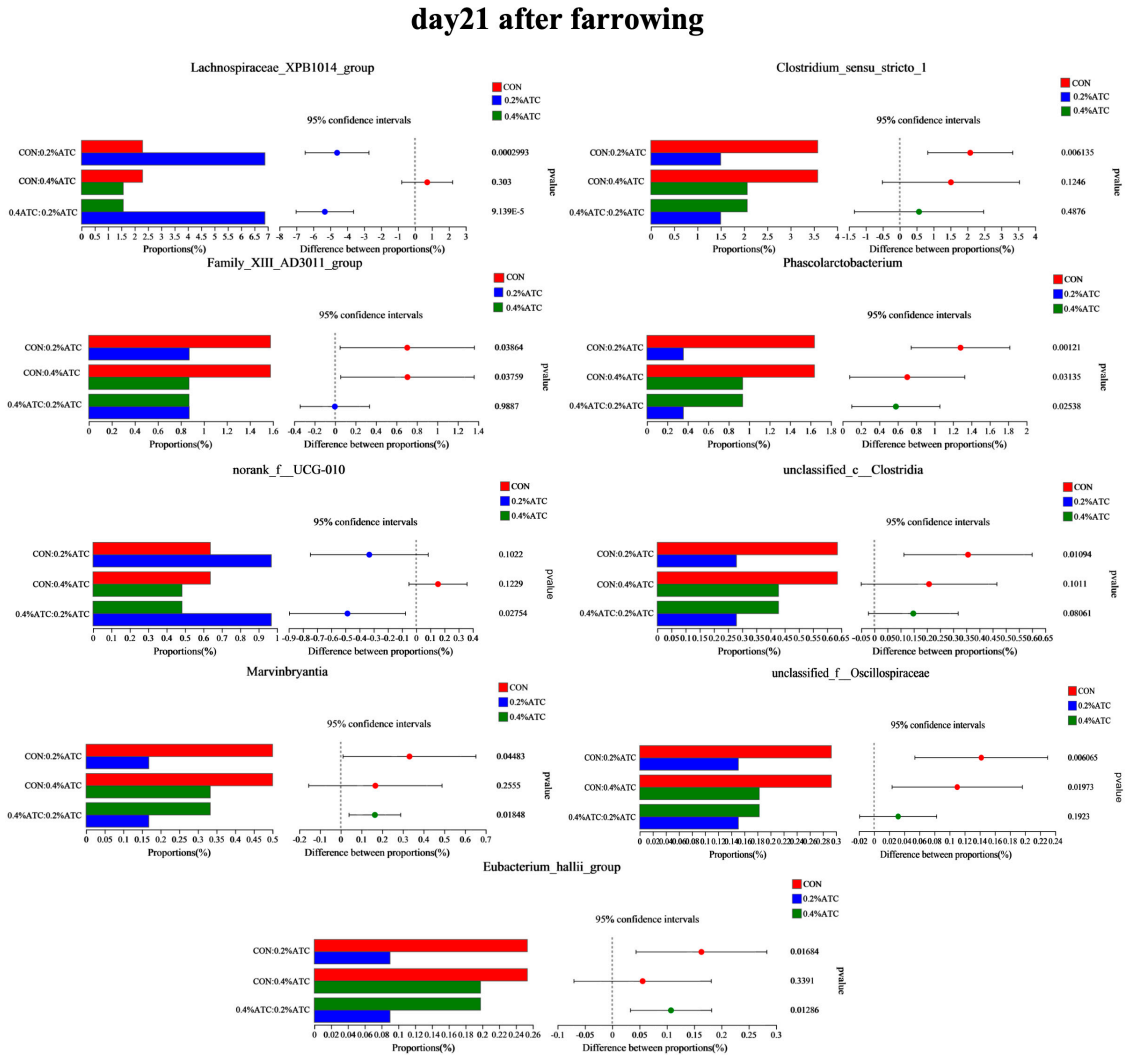
Changes in bacterial genera of gut microbiota of sows on day 21 postpartum.

The intestinal flora composition of sows in the different groups was analyzed at the phylum and genus levels. No significant differences were observed at the phylum level among the groups on day 1 postpartum (*p* > 0.05). As shown in Figure 9A, supplementation with 0.2% ATC increased the relative abundance of Cyanobacteria on day 21 postpartum (*p* < 0.05). At the genus level, as shown in Figure 9B, on day 1 postpartum, 0.2% ATC supplementation increased the relative abundance of *norank_f__Eubacterium_coprostanoligenes group* (*p* < 0.05), while 0.4% ATC increased the relative abundance of *norank_f__Eubacterium_coprostanoligenes group*, *Blautia*, and *Coprococcus* (*p* < 0.05). As shown in Figure 10, on day 21 postpartum, 0.2% ATC supplementation increased the relative abundance *of Lachnospiraceae_XPB1014 group* (*p* < 0.05) but decreased the relative abundance of *Clostridium_sensu_stricto_1*, *Family_XIII_AD3011 group*, *Phascolarctobacterium*, *unclassified_c__Clostridia*, *unclassified_f__Oscillospiraceae*, and *Eubacterium_hallii group* (*p* < 0.05). Additionally, 0.4% ATC supplementation decreased the abundance of *Family_XIII_AD3011 group*, *Phascolarctobacterium*, and *unclassified_f__Oscillospiraceae* (*p* < 0.05).

### 3.9. Spearman’s Correlation among Gut Microbiota and Serum Immune Parameters

**Figure 11:**
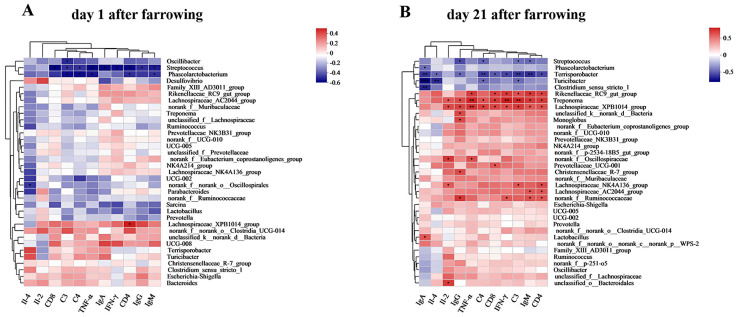
Spearman correlation heatmap at genus level. (**A**) Spearman correlation heatmap at genus level of sows on day 1 postpartum. (**B**) Spearman correlation heatmap at genus level of sows on day 21 postpartum. Note: “*” indicates significant difference (*p* < 0.05), “**” indicates highly significant difference (*p* < 0.01), “***”indicates very highly significant difference (*p* < 0.001) of spearman correlation heatmap.

As shown in Figure 11A, *Oscillibacter* was negatively correlated with C3 (*p* < 0.05), *Streptococcus* was significantly negatively correlated with serum CD8, C3, C4, TNF-α, IgA, IFN-γ, CD4, IgG, IgM (*p* < 0.05), and *Phascolarctobacterium* was negatively correlated with C3, C4, TNF-α, CD4, IgM (*p* < 0.05). *Lachnospiraceae_XPB1014 group* was positively correlated with CD4 (*p* < 0.05).

As shown in Figure 11B, *Streptococcus* was negatively correlated with IgG, C4, C3 and IgM (*p* < 0.05), and *Phascolarctobacterium* was negatively correlated with IgA (*p* < 0.05). *Terrisporobacter* was negatively correlated with IgA, Il-4, IgG, C4, CD8, IFN-γ, C3, IgM, and CD4 (*p* < 0.05). *Turicibacter* was negatively correlated with IgA, Il-4, C4, and C3 (*p* < 0.05). *Clostridium_sensu_stricto_1* was negatively correlated with IgA (*p* < 0.05). *Rikenellaceae_RC9_gut group* was negatively correlated with TNF-α, CD8 and IFN-γ, C3, IgM, and CD4. *Treponema* was positively correlated with Il-2, IgG, TNF-α, C4, CD8, IFN-γ, C3, IgM, and CD4 (*p* < 0.05). *Lachnospiraceae_XPB1014 group* was positively correlated with IL-2, IgG, TNF-α, C4, CD8, IFN-γ, C3, IgM, and CD4 (*p* < 0.05). *norank_f_Oscillospiraceae* was positively correlated with Il-2 and TNF-α (*p* < 0.05). *Lachnospiraceae_NK4A136 group* was positively correlated with Il-2, C3, and CD4 (*p* < 0.05). *Lachnospiraceae_AC2044 group* was positively correlated with IgM and CD4 (*p* < 0.05). *Norank_f_Ruminococcaceae* was positively correlated with IgG, IFN-γ, IgM, and CD4 (*p* < 0.05). *Unclassified_k_norank_d_Bacteria*, *Monoglobus*, and *Christensenellaceae_R-7 group* were positively correlated with IgG (*p* < 0.05). *Prevotellaceae_UCG-001*, *Lactobacillus*, and *unclassified_o_Bacteroidales* were significantly positively correlated with CD8, IgA, and Il-2, respectively (*p* < 0.05).

## 4. Discussion

Feed intake is widely recognized as a crucial factor impacting the overall performance of the sows. Inadequate feed intake during lactation can result in weight loss and reduced milk production in sows [21]. In our study, we observed that the inclusion of 0.2% ATC in the sow’s diet did not exert a significant influence on the average daily feed intake from day 1 to 21 postpartum. Similar findings were reported in a study where the supplementation of 1 g ATC/kg ATC in the basal diet of piglets did not increase the average daily feed intake [22]. However, it is important to note that research outcomes in this regard are not always consistent. For instance, a study investigating the supplementation of 50 mg/kg ATC in the diet of weaned calves reported that ATC supplementation increased body weight, feed efficiency, and average daily weight gain [20]. Such inconsistencies might arise from variations in nutrient absorption and utilization efficiency across different animal species, physiological states, and experimental conditions.

Our study indicates that supplementing sows’ diets with 0.2% and 0.4% ATC boosted average daily milk production by 4.98% and 9.38%, respectively. While not reaching statistical significance compared to the CON group, there was a trend toward enhanced milk production in the sows. To ascertain the effect of ATC on sow milk production, further validation is warranted in subsequent experiments. While there is limited research investigating the impact of ATC on sow lactation, studies focusing on dairy cows have yielded varying conclusions. For example, the supplementation of 30 g/d ATC in the diet of Holstein cows increased daily milk yield from 22.5 kg/d to 24 kg/d [23]. Similarly, supplementation of 30 g/d, 60 g/d, and 300 g/d ATC in the mixed diet of dairy cows exhibited a linear increase in milk yield. The observed increase in milk yield in dairy cows can be attributed to the reduction in mastitis occurrence caused by oxidative stress during the perinatal period, which is mitigated by ATC supplementation. ATC culture has been shown to enhance antioxidant and immune performance in dairy cows, leading to improved rumen fermentation environment, reduced mastitis incidence, and enhanced stability of rumen metabolism [24].

Sow milk serves as the primary source of nutrition for piglets, with a significant transfer of at least 50% of nitrogen and energy from the sow’s diet to the piglets occurring through milk during peak lactation [25]. Our study findings demonstrated that the supplementation of 0.2% ATC resulted in increased fat and total solid (TS) content in the sow milk on day 21 postpartum. Milk fat is a highly variable component in pig milk and is particularly responsive to changes in the sow’s dietary nutrition [11]. PRL and its receptor can activate intracellular tyrosine kinase (JAK2), thereby regulating the expression of milk fat synthesis-related enzyme genes through the JAK–STAT signaling pathway. This regulation influences the synthesis and activity of milk fat synthesis-related enzymes, ultimately impacting milk fat synthesis [26]. Consequently, the observed increase in fat content in sow milk on day 21 postpartum may be attributed to the enhanced secretion of PRL. In studies involving dairy cows, ATC supplementation in the mixed diet increased milk fat content [23].

*Cordyceps sinensis* is recognized as a tonic with potential benefits for energy, endurance, and libido. Its effects on sex hormones and energy metabolism have been investigated. For instance, cordycepin has been found to inhibit PRL secretion in rat pituitary tumor cells (GH3) by inducing the expression of cell surface protein ADORA1 and regulating the activation of p-PI3K, p-AKT, p-ERK1/2, and other proteins [27]. The regulation of *Cordyceps* on hormones has been reported in male animals such that the serum testosterone (T) and E2 concentrations of rats fed with *Cordyceps militaris* increased, but the follicle stimulating hormone (FSH), luteinizing hormone (LH), or PRL and other hormones did not increase [28]. PRL and GH play important roles in regulating substance metabolism, ensuring nutrient absorption by mammary cells for milk formation, and participating in the synthesis and secretion of thymosin by the thymus epithelial cells, thereby indirectly modulating immune function [29]. In our study, the supplementation of 0.2% ATC increased the PRL content in the sow serum at both 1 day and 21 days postpartum, whereas the GH levels did not exhibit significant changes. These findings suggest that PRL may not primarily regulate lactation through a synergistic action with GH. Consequently, the observed increase in PRL content in our study may be attributed to the effects of cordycepic acid, *Cordyceps* polysaccharide, and other components present in ATC. Moreover, the supplementation of 0.2% ATC resulted in elevated serum concentrations of triiodothyronine (T3), thyroxine (T4), and E2. Thyroid hormones influence cardiovascular hemodynamics, cardiac filling, and myocardial contractility [30,31]. T3, in particular, can affect cardiac function by binding to specific nuclear receptors and regulating the transcription of various genes involved in cardiovascular function [31]. Simultaneously, T3 regulates peripheral circulation and influences milk composition synthesis. Higher levels of thyroid hormones promote breast blood circulation, enhance the metabolic rate and function of breast acinar cells. E2, as the most abundant and biologically active substance in the body, promotes the development of the female reproductive tract, proliferation of vaginal epithelial cells, increases the sensitivity of uterine smooth muscle to oxytocin, and cooperates with PRL in maintaining lactation. Overall, our results indicate that ATC supplementation in sow diets can stimulate the secretion of certain serum hormones, acting to influence sow lactation. The effects on hormone secretion are more pronounced at 21 days postpartum compared to 1 day postpartum. Furthermore, compared with supplementing 0.4% ATC, supplementing 0.2% ATC has a more significant promoting effect on PRL, T3, T4, and E2 in the serum of postpartum sows at 21 days.

Immunoglobulins are a type of globulin produced in response to antigen stimulation in animals. They possess biological functions such as antigen binding and complement activation. IgM is the first immunoglobulin secreted in the body but has a short duration. It plays a role in pathogen dissolution by binding with complements when pathogens are present. IgG, one of the main immunoglobulins found in serum, agglutinates or precipitates antigens, thereby regulating antibacterial, antiviral, and other immune activities [32]; it holds significant importance in humoral immunity. IgA acts by directly neutralizing or preventing the binding of toxins, viruses, and bacteria to mucosal surfaces [33]. Our findings revealed that supplementation with 0.2% ATC increased the serum levels of IgA, IgM, and IgG on the first day postpartum. These results are consistent with the immunomodulatory effects of ATC observed in Holstein weaned calves and adult Holstein weaned calves [20,23]. These findings suggest that ATC supplementation can enhance the secretion of animal immunoglobulins and improve immune function.

Our results indicated that the supplementation of 0.4% ATC reduced the serum levels of IL-2 on day 21 postpartum. IL-2 is recognized as a proinflammatory cytokine, and its decreased production can protect organisms from excessive inflammatory responses [34]. This effect may be attributed to cordycepin, which has been shown to significantly inhibit the overproduction of pro-inflammatory cytokines in a concentration-dependent manner without causing cytotoxicity [35]. CD4 cells, a subset of T lymphocytes, play a crucial role in activating lymphocyte activity and initiating cellular immunity through signal transduction. On the other hand, CD8 cells are involved in target cell dissolution, immune regulation, and the inhibition of cellular and humoral immunity. Through cytotoxic effects, CD8 cells can induce cell apoptosis and contribute to a decrease in immune function. Our findings revealed that the supplementation of 0.2% ATC increased the serum levels of CD4 and CD8 on both day 1 and 21 postpartum. However, ATC supplementation did not alter the CD4/CD8 ratio in the serum of sows, suggesting that ATC did not affect the dynamic balance between CD4 and CD8 subsets. Furthermore, the supplementation of 0.2% ATC also elevated the serum content of IFN-γ on day 21 postpartum. IFN-γ is primarily produced by natural killer cells (NK) and natural killer T cells (NKT) as part of the innate immune response. It is also produced by CD4, Th1, and CD8 cytotoxic T lymphocyte (CTL) effector T cells during adaptive immunity [36]. In summary, the supplementation of ATC enhanced the immune function of lactating sows.

Cordycepic acid, cordycepic polysaccharide, and ergosterol, the primary constituents of ATC, have been demonstrated to possess notable antioxidant and antitumor potential in various forms of tumors [37,38]. Our investigation has ascertained that the introduction of a 0.2% ATC supplement resulted in a decrease in the serum levels of SOD and CAT on the initial day following parturition, while it posed no significant impact on MDA, GSH-Px, and T-AOC levels in sow serum. The antioxidant capabilities of ATC have been exhibited in bovine dietary applications, as well as in other species such as poultry and shrimp. Intake of *Cordyceps* mycelial polysaccharides notably escalates the activity of SOD, GSH-x, ROS, T-AOC, along with other antioxidant indices in prawns [39]. Additionally, the introduction of 3 g/kg and 5 g/kg of ATC to the basal diet notably mitigated the content of alanine ALT in the serum of female geese, significantly amplified the T-AOC levels within the serum, and notably reduced the MDA content in the serum [40]. In our current investigation, the application of ATC failed to present discernible effects in the enhancement of sows’ antioxidant capacity. The disparities in results might be attributed to variations in the animal species or the short duration of ATC action from feeding to the point of one day post-sow delivery. The sow’s physiological condition on the first day after delivery, which is unique, may also play a role. In related research conducted on piglets, the supplementation of the basal diet with 1 g ATC/kg resulted in an elevation in the levels of blood GSH, GSH-Px, and CAT [22]. We assume that these contrasting results could be attributed to the underdeveloped intestinal barrier in piglets alongside the postpartum stress experienced by sows. It reminds us that we need to comprehensively and more accurately understand the effect of ATC on the antioxidant capacity of sows and its potential mechanism.

Intestinal flora plays an important role in nutrition and disease prevention. It is responsible for metabolizing undigested polysaccharides, resistant starch, and fibers, leading to the production of short-chain fatty acids (SCFAs) that exert various health effects [41]. Additionally, intestinal bacteria contribute to vitamin synthesis and influence the host’s immune response. The diversity of intestinal microbiota has emerged as a novel biomarker of health and metabolic capacity, as low microbial diversity is often associated with conditions such as inflammation, oxidative stress, and obesity [42,43,44]. Our findings demonstrated that the supplementation of ATC had an impact on the β diversity of sow intestinal flora on day 21 postpartum. It is worth noting that the phylogenetic composition of gut microbiota in sows undergoes significant changes during pregnancy and lactation. Previous studies have indicated that Firmicutes and Bacteroidota are the predominant phyla across all reproductive stages of sows [45]. Specifically, our results revealed that Firmicutes and Bacteroidetes were the dominant intestinal flora in sows on day 21 postpartum, whereas Firmicutes and Proteobacteria were dominant on day 1 postpartum. The reason for the change in the dominant intestinal flora of sows on day 1 postpartum may be that sows have a metabolic syndrome and mild inflammation in the perinatal period, especially in the early lactation period [46], while Proteobacteria will multiply during low-level intestinal inflammation. For instance, the relative abundance of Proteobacteria in the gut of sows on day 3 of lactation was higher than that on day 30 of pregnancy, day 109 of pregnancy, and day 14 of lactation [46]. We assume that as pregnancy progresses, the relative abundance of Proteobacteria in the gut microbiota of sows increases, potentially promoting inflammatory responses.

The composition and metabolic activities of intestinal flora are primarily influenced by dietary factors. The type and quantity of diet, as well as the balance of major nutrients, exert a substantial impact on the microbial population within the intestine. Notably, cordycepin has been shown to modulate the distribution of microorganisms within the host by targeting cell adenosine kinase (AdoK) [47]; furthermore, cordycepin polysaccharide has demonstrated the ability to stimulate the proliferation of bifidobacterium colonies in vitro and enhance acetic acid production [48].

Our results showed that the supplementation of 0.2% ATC increased the relative abundance of *norank_f__Eubacterium_coprostanoligenes group*. The supplementation of 0.4% ATC increased the relative abundance of *Blautia*, *norank_f__Eubacterium_coprostanoligenes group*, and *Coprococcus*. The relative abundance of *norank_f__Eubacterium_coprostanoligenes group*, and *Blautia* had a significant negative correlation with the content of LPS [49]. LPS is involved in the key inflammatory signal transduction pathway of ulcerative colitis, which can mediate inflammatory response and promote the synthesis of inflammatory factors, leading to the further deterioration of epithelial barrier dysfunction. While the relative abundance of *norank_f__Eubacterium_coprostanoligenes group* was negatively correlated with the disease activity index of ulcerative colitis [50]. As a symbiotic obligate anaerobic bacterium, *Blautia* plays an important role in preventing inflammation by upregulating intestinal Treg [51]. *Blautia* has been associated with reduced mortality and improved overall survival from acute graft-versus-host disease [52]. *Blautia* may be able to regulate the level of citrulline in the blood, which has been identified as a biomarker of intestinal barrier integrity and function, and its low level in the blood is associated with bacteremia and inflammation [53]. Moreover, *Blautia*, as a prevalent producer of acetic acid in the intestine, exerts its effects by activating G-protein-coupled receptors GPR41 and GPR43, which in turn inhibit the INS signaling pathway and adipocyte fat accumulation. Consequently, this metabolic activity contributes to the alleviation of obesity-related disorders [54]. Additionally, *Blautia* is involved in glucose metabolism, generating acetate, lactate, succinate, ethanol, and hydrogen as major metabolites. It also exhibits antimicrobial properties by producing bacteriocins, which help regulate the intestinal microbiota and prevent colonization by pathogens [55,56,57]. Our experiment showed that the addition of ATC could alleviate animal intestinal inflammation by increasing the relative abundance of beneficial bacteria.

At the phylum level, our results indicated that the supplementation of 0.2% ATC led to an increased relative abundance of Cyanobacteria on day 21 postpartum. Cyanobacteria, considered the oldest Gram-negative bacteria and oxygenic photosynthetic bacteria, are known for their ability to enhance nitrogen fixation in various ecological environments. In addition to their photosynthetic capabilities, members of cyanobacteria are involved in the synthesis and catabolism of glutathione, thereby influencing the degradation of harmful substances and potentially exerting beneficial effects [58]. Interestingly, the relative abundance of cyanobacteria showed a negative correlation with the levels of inflammatory cytokines, such as IL-6 and TNF-α, while the anti-inflammatory factor IL-10 exhibited a positive correlation [59]. These findings suggest that Cyanobacteria may possess anti-inflammatory properties and potentially contribute to the inhibition of intestinal inflammation.

The supplementation of 0.2% ATC resulted in an increased relative abundance of *Lachnospiraceae_XPB1014 group*. Our study further revealed a positive correlation between the relative abundance of *Lachnospiraceae_XPB1014 group* and the levels of IgG, IgM, IL-2, C3, C4, CD4, CD8, and IFN-γ in the serum of sows, as determined through Spearman’s correlation analysis. Consistently, the supplementation of 0.2% ATC also led to increased serum levels of immunoglobulins and complement factors in the sows. These findings suggest that the notable enrichment of *Lachnospiraceae_XPB1014 group* may contribute to the enhancement of immune function in sows.

The supplementation of ATC reduced the relative abundance of *Phascolarctobacterium*. *Phascolarctobacterium* is a pro-inflammatory bacterium that is related to the occurrence and development of inflammation in metabolic diseases and mental diseases [60,61]. Enrichment of *Phascolarctobacterium* in the shrimp gut may lead to the occurrence of white stool syndrome [62]. In addition, the relative abundance of *Phascolarctobacterium* in kidney stone patients was higher than that in healthy controls [63]. Our study also showed that *Phascolarctobacterium* was negatively correlated with the IgM, C3, C4, and CD4 levels, resulting in decreased immune function. In the study of animal intestinal flora, *Clostridium_sensu_stricto_1* is usually considered to be a pathogen, and there is research evidence that it is closely related to inflammation [64]. The enrichment of *Clostridium_sensu_stricto _1* was positively correlated with the mRNA expression of TNF-α and IL-1β, which eventually led to colonic inflammation [65]. Moreover, increased relative abundance of *Clostridium_sensu_stricto_1* was observed in other inflammatory models, such as endometritis [66], necrotizing enterocolitis [67] and LPS-induced intestinal inflammation [68]. *Clostridium_sensu_stricto_1* is a member of the *Clostridium* family. *Clostridium* is the most important pathogen of various human diseases, such as sudden death, toxicity, mutation, carcinogenesis or aging. It biotransforms various ingested or endogenously formed compounds into harmful substances such as N-nitroso compounds or aromatic steroids in the gastrointestinal tract [69]. These findings collectively indicate that ATC supplementation promotes the enrichment of beneficial bacteria while reducing the relative abundance of harmful bacteria, thereby modulating the composition of the intestinal flora. By exerting these effects, ATC has the potential to positively influence the intestinal microbiota and contribute to the regulation of gut microbial balance.

## 5. Conclusions

The addition of ATC to the late gestation and lactation diets of sows has been shown to elicit several beneficial effects. It has the effect of increasing thyroid-related hormones, specific antibodies, and cytokines in serum, as well as a tendency to enhance milk production in sows. Furthermore, ATC supplementation has the ability to modulate the structure of the intestinal flora in sows by increasing the abundance of beneficial bacteria while reducing the abundance of harmful bacteria. In our study, the optimal supplemental dosage of ATC in the late gestation diet of sows was determined to be 0.2%. These findings highlight the potential of ATC as a valuable dietary intervention for optimizing sow health and performance during this critical period.

## Figures and Tables

**Table 1:** Composition and nutrient levels of diets for sows.

Composition	Component (%)	Nutrient Level	Content
Corn	62	Digestive energy, MJ/kg	13
Soybean meal	25	Crude protein, %	17.1
Wheat bran	4	Total calcium, %	0.7
Steamed fish meal	1	Total phosphorus, %	0.6
Fat powder	4	Lysine, %	0.75
Premix *	4	Methionine, %	0.23
Total	100	Threonine, %	0.5

*: Ingredients per kg premix: vitamin A, 7000 IU; vitamin D, 500 IU; vitamin E, 65 IU; vitamin K, 4 mg; vitamin B2, 5 mg; vitamin B3, 30 mg; vitamin B12, 0.03 mg; pantothenic acid, 20 mg; choline, 350 mg; folic acid, 0.65 mg; biotin, 2 mg; copper, 20 mg; iron, 110 mg; zinc, 100 mg; manganese, 40 mg; iodine, 0.4 mg; selenium, 0.4 mg.

**Table 2:** Effects of ATC on daily feed intake of lactation sows (kg/d).

Daily Feed Intake	CON	0.2% ATC	0.4% ATC
1–7 d	3.94 ± 0.13	4.20 ± 0.133	4.11 ± 0.15
8–14 d	5.83 ± 0.09 ^b^	6.12 ± 0.08 ^a^	5.92 ± 0.08 ^ab^
15–21 d	6.28 ± 0.09	6.31 ± 0.067	6.16 ± 0.08
1–21 d	5.35 ± 0.08	5.54 ± 0.08	5.40 ± 0.08

Note: Peer data shoulders marked with different lowercase letters indicate significant differences (*p* < 0.05).

**Table 3:** Effects of ATC on average daily milk production of sows (kg/d).

Daily Milk Production	CON	0.2% ATC	0.4% ATC
1–7 d	7.00 ± 0.42	7.26 ± 0.50	7.20 ± 0.48
8–14 d	9.30 ± 0.65	9.49 ± 0.64	10.86 ± 0.63
15–21 d	9.58 ± 0.47	10.41 ± 0.63	10.26 ± 0.50
1–21 d	8.63 ± 0.34	9.06 ± 0.39	9.44 ± 0.39

**Table 4:** Effects of ATC on milk composition of sows.

Items	Day 1 Postpartum			Day 21 Postpartum		
	CON	0.2% ATC	0.4% ATC	CON	0.2% ATC	0.4% ATC
Fat (%)	4.95 ± 0.43	4.99 ± 0.38	5.33 ± 0.68	6.86 ± 0.57 ^b^	10.93 ± 1.43 ^a^	8.67 ± 0.79 ^ab^
Protein (%)	14.37 ± 0.81	14.45 ± 0.69	13.43 ± 0.79	5 ± 0.28	5.4 ± 0.21	5.21 ± 0.11
Lactose (%)	3.02 ± 0.09	2.9 ± 0.08	2.76 ± 0.11	5.39 ± 0.49	5.43 ± 0.15	5.74 ± 0.11
TS (%)	25.76 ± 1.14	26.85 ± 0.74	25.66 ± 1.67	18.36 ± 0.58 ^b^	22.34 ± 1.47 ^a^	20.72 ± 0.72 ^ab^
SNF (%)	19.74 ± 0.87	21.02 ± 0.57	20.16 ± 1.19	11.42 ± 0.27	11.68 ± 0.08	11.98 ± 0.15
Density (g/L)	1050.18 ± 1.77	1052.49 ± 1.53	1050.1 ± 2.97	1036.13 ± 2.18	1031.55 ± 2.33	1036.52 ± 1.54
Acidity (°T)	50.88 ± 3.13	54.1 ± 2.26	50.71 ± 4.01	8.97 ± 1.12	8.37 ± 0.41	8.61 ± 0.17
FPD (°C)	−1.21 ± 0.06	−1.26 ± 0.03	−1.19 ± 0.067	−0.78 ± 0.02 ^b^	−0.87 ± 0.02 ^a^	−0.84 ± 0.02 ^ab^
FFA (mg/L)	62.89 ± 3.63	66.8 ± 2.15	59.78 ± 5.05	24.42 ± 1.33	24.16 ± 1.36	26.03 ± 1.35
CA (%)	0.2 ± 0.01	0.18 ± 0.01	0.21 ± 0.02	0.11 ± 0.01	0.11 ± 0.01	0.12 ± 0.01

Note: Peer data shoulders marked with different lowercase letters indicate significant differences (*p* < 0.05).

## Data Availability

The original contributions presented in the study are included in the article. further inquiries can be directed to the corresponding author.
